# Folic-acid-conjugated pullulan/poly(DL-lactide-co-glycolide) graft copolymer nanoparticles for folate-receptor-mediated drug delivery

**DOI:** 10.1186/s11671-014-0706-1

**Published:** 2015-02-06

**Authors:** Sang Joon Lee, Yong-Ho Shim, Jong-Suk Oh, Young-Il Jeong, In-Kyu Park, Hyun Chul Lee

**Affiliations:** Department of Biomedical Sciences, Chonnam National University Medical School, Gwangju, 501-746 Korea; Biomedical Research Institute, Pusan National University Hospital, Pusan, 602-739 Republic of Korea; Department of Microbiology, Chonnam National University Medical School, Gwangju, 501-746 Korea

**Keywords:** Folate receptor, Pullulan, Nanoparticles, KB cells, Drug targeting

## Abstract

**Background:**

Nanoparticles have been extensively investigated for targeted delivery of anticancer drugs. Since the folate receptor is universally over-expressed on the tumor cell membrane, folic acid is often used to modify the fate of nanoparticles in biologicals.

**Methods:**

To fabricate targetable nanoparticles, folic acid was conjugated to a pullulan backbone and poly(DL-lactide-co-glycolide) (PLGA) (abbreviated as FAPuLG) was conjugated. KB cells and NIH3T3-cell-bearing mice were prepared to prove folate receptor targeting of FAPuLG nanoparticles.

**Results and discussion:**

Nanoparticles of FAPuLG copolymer that self-assembled in water were small with diameters <200 nm. Doxorubicin (DOX) as a model drug was incorporated into the FAPuLG nanoparticles that were used to treat folate receptor over-expressing KB human carcinoma cells. Fluorescence microscopy revealed that DOX-incorporated FAPuLG nanoparticles induced strong red fluorescence in the KB cells in the absence of folic acid. However, fluorescence intensity was decreased by blocking folate receptors. Antitumor activity of FAPuLG nanoparticles against KB cells *in vitro* was also decreased by blocking folate receptors. In animal study using near-infrared dye-conjugated FAPuLG nanoparticles, fluorescence intensity was significantly higher at KB solid tumor than that of NIH3T3.

**Conclusions:**

The results indicate that FAPuLG nanoparticles can target the folate receptor of tumor cells. FAPuLG nanoparticles are a promising candidate for active targeting of anticancer agents.

## Background

Nanoparticle-based targeted drug delivery systems have been extensively investigated for biomedical applications [1-4]. The unique features of nanoscale carriers are their huge surface area versus volume, ease of surface modification, prolonged circulation in the blood through avoidance of the reticuloendothelial system, and small size that facilitates specific interaction with cell surface receptors, enabling the targeting of specific cells or tissues having disease in the specific organs [1,2,5-10]. Furthermore, tumor-specific targeting of nanoparticles allows the killing of tumor cells selectively without harm to healthy tissues or cells [6-8].

Since nanoparticles themselves do not possess tumor specificity, various kinds of tumor-specific ligand such as endothelial growth-factor-related peptide [5], hyaluronic acid [6], galactose [7], folic acid [8], monoclonal antibody [9], and transferrin [10] have been used to bestow tumor-specific drug targeting of nanoparticles. Among them, the folate receptor is over-expressed in virtually all types of tumor cells, while being barely detectable in healthy tissues and cells [11-13]. Folic-acid-anchored nanoparticles have tumor-specific targeting of anticancer drug via folate receptors [13,14]. One study reported that doxorubicin (DOX)-encapsulated nanoparticles having folic acid on the particle surface were remarkably efficient for the regression of tumor growth and reduction of toxicity compared to free DOX or plain nanoparticles [13]. Furthermore, nanoparticles having folic acid as a targeting moiety facilitate accumulation of anticancer agents in tumors [14].

In this study, folic-acid-conjugated pullulan-g-poly(DL-lactide-co-glycolide) (PLGA) (abbreviated as FAPuLG) copolymers were synthesized for targeted delivery of a model anticancer agent (DOX) to tumor cells. The advantages of pullulan include biocompatibility, immuno-neutrality, and numerous functional hydroxyl groups [15,16]. Hence, pullulan can be used as a drug-carrying material and its derivatives, such as pullulan/PLGA graft copolymer, can be considered as an ideal vehicle for fabrication of drug-targeting carriers.

We previously reported that PuLG copolymer can self-assemble in an aqueous environment to form small nanospheres <100 nm in diameter, which are useful as a drug-delivery carrier [17]. Physicochemical properties of nanoparticles of FAPuLG were studied and their targeting potential was assessed using KB tumor cells *in vitro*.

## Methods

### Materials

Pullulan (M_n_ 67,500) and PLGA 5005 (M_n_ 8,200) were purchased from Wako Pure Chemical Industries, Osaka, Japan. Folic acid, N,N′-dicyclohexyl carbodiimide (DCC) and 4-(N,N-dimethylamino)pyridine (DMAP) were purchased from Aldrich Chemical (St. Louis, MO, USA). DOX HCl and triethyleamine (TEA) were purchased from Sigma-Aldrich (St. Louis, MO, USA). Spectra/Pro™ dialysis membranes with a molecular weight cutoff (MWCO) of 6,500 g/mol were purchased from Spectrum Laboratories (Rancho Dominguez, CA, USA). Sulfo-cyanine 5 carboxylic acid was purchased from Lumiprobe Co. (Florida, USA). Dichloromethane (DCM) and dimethyl sulfoxide (DMSO) of high-performance liquid chromatography grade were dried with CaH2 then distilled prior to use. ^1^H nuclear magnetic resonance (NMR) spectra were recorded in deuterated solvents (DMSO-d6) at a concentration of 30 mg/0.6 ml using a model AMX-300 apparatus (Bruker, Billerica, MA, USA).

### Synthesis of folic-acid-conjugated copolymer

Pullulan (675 mg) was dissolved in DMSO (15 ml) for 3 h. Folic acid was dissolved in DMSO (5 ml) with a 1.1 equivalent amount of DCC and DMAP for 30 min. Folic acid solution was added into the pullulan solution and stirred for 24 h in a nitrogen atmosphere. The resulting solution was filtered to remove by-products and dialyzed against deionized water for 3 days, followed by lyophilization. The synthesized folic acid-pullulan (FA-PU) conjugates were analyzed with NMR. FA-Pu-PLGA (abbreviated as FAPuLG) copolymers were synthesized as follows. Three hundred milligrams of FA-PU and 182 mg of PLGA were dissolved separately in dried DMSO. A 1.2 equivalent amount DCC in DMSO solution was added to the PLGA/DMSO solution, which was stirred for 30 min to activate the carboxyl group of the PLGA. The resulting solution was added to the pullulan/DMSO solution containing 1.2 equivalent amount DMAP, and the reaction was allowed to continue at room temperature for 24 h. The reaction mixture was filtered to remove the by-products and exhaustively dialyzed against deionized water for 3 days. The dialyzed solution was lyophilized for 3 days, and the solid product was precipitated three times in DCM to remove unreacted PLGA and dried in a vacuum oven for 2 days. The substitution degree of folic acid and PLGA was calculated from molecular weight of pullulan and PLGA [17]. PuLG copolymer was synthesized as described previously [17]. The number-average molecular weight (Mn) of synthesized copolymers was analyzed using ^1^H NMR spectroscopy. The molecular weight (M.W.) of pullulan and PLGA was analyzed using gel permeation chromatography as reported previously [17]. The weight average M.W. (Mw), Mn, and polydispersity of pullulan were 67,500, 62,400, and 1.081, respectively. The Mw, Mn, and polydispersity of PLGA were 8,200, 5,800, and 1.41, respectively.

Near-infrared absorption dye (NIR dye) conjugation was performed as follows: 20 mg of FAPuLG copolymer was dissolved in dry DMSO. To this solution, 1 mg of sulfo-cyanine 5 carboxylic acid with equivalent mole of DCC and DMAP was added and then stirred for 12 h in the room temperature. After that, resulting solution was dialyzed to remove unreacted dye with exchange of water at intervals of 3 h. The dialysis procedure was continued until that dye was not detected. Dye in the dialyzed solution was detected with UV spectrophotometer (UV-1601 UV-VIS spectrophotometer, Shimadzu, Kyoto, Japan) at 642 nm. To evaluate conjugation yield, 3 mg of dye-conjugated polymer was dissolved in DMSO and then the contents of the dye in the polymer were measured with the UV spectrophotometer. For a blank test, polymer without dye was used. Dye contents (%, *w*/*w*) = [(Weight of dye in the polymer/total weight of polymer) × 100]. The content of the dye in the polymer was approximately 3.2% (*w*/*w*).

### Preparation of nanoparticles

Twenty milligrams of PuLG or FAPuLG was dissolved in 3 ml of DMSO. Five milligrams of DOX was separately dissolved in 1 ml of DMSO with trace amounts of TEA and added to the polymer/DMSO solution. The resulting solution was slowly added dropwise to 10 ml of deionized water for 10 min and then loaded into dialysis tubing. The tubing was put into 1 l of deionized water. During dialysis, DOX-loaded nanoparticles formed and organic solvent was removed. Dialysis was continued for 12 h with changes of the water every 2 h. The resulting solution was analyzed or lyophilized. To determine drug contents, 5 mg of lyophilized nanoparticles were dissolved in DMSO, and the absorbance was measured using a UV spectrophotometer (UV-1601 UV-VIS spectrophotometer, Shimadzu, Kyoto, Japan) at 479 nm. Drug contents (%, *w*/*w*) were calculated as (amount of DOX in the nanoparticles/weight of nanoparticles) × 100.

### Characterization of nanoparticles

Nanoparticle morphology was performed using transmission electron microscopy (TEM) using a JEM-2000 FX II microscope (JEOL, Tokyo, Japan). The diameter of the nanoparticles was measured with a model DLS-7000 dynamic laser scattering apparatus (Otsuka Electronics Company, Osaka, Japan). A sample solution prepared by dialysis was used to determine particle size (concentration 1 mg/ml). The critical association concentration (CAC) of nanoparticles was evaluated with a model RF-5301 fluorescence spectrofluorophotometer (Shimadzu, Tokyo, Japan) as described previously [17]. PuLG or FAPuLG nanoparticles in the absence of a drug were prepared as described above. Polymer (40 mg) was dissolved in DMSO (5 ml) and dialyzed against deionized water for up to 2 days. The nanoparticle suspension was adjusted to various concentrations. The CAC of nanoparticles was estimated using pyrene as a hydrophobic probe. To prepare sample solutions, a known amount of pyrene in acetone was added to each of a series of 20-ml vials, and the acetone was evaporated. The final concentration of pyrene was 6.0 × 10^−7^ M. To each vial, 10 ml of various concentrations of the nanoparticle suspensions was added, and the solutions were heated for 3 h at 65°C. Equilibration of the pyrene and the PuLG nanoparticles was achieved by allowing the solutions to cool overnight at room temperature. The fluorescence excitation spectra were measured at an emission wavelength of 390 nm. Excitation and emission bandwidths were 1.5 and 1.5 nm, respectively.

### Drug release study

Drug release from nanoparticles was carried out at physiological solutions. Five milligrams of nanoparticle solid was reconstituted into 5 ml of deionized water and then introduced into a dialysis tubing. The sealed dialysis tubing was added to a 50-ml Falcon tube containing 40 ml of phosphate-buffered saline (PBS) solutions (pH 7.4, 0.1 M). These tubes were placed in a shaking incubator with a stirring speed of 100 rpm at 37°C. At specific times, the medium was sampled for analysis of drug concentration. Afterwards, the entire medium was replaced with fresh medium to prevent drug saturation. The concentration of drug released was measured using the aforementioned ultraviolet spectrophotometer at 479 nm.

### Cell culture

KB human oral squamous carcinoma cells and NIH3T3 mouse fibroblast cells were obtained from the Korean Cell Line Bank (KCLB, Seoul, Korea) and maintained with DMEM medium supplemented with 10% fetal bovine serum (FBS) and 1% antibiotics in a 5% CO_2_ incubator operating at 37°C.

### Targetability of nanoparticles for folate receptor of KB cell

To study targetability of nanoparticles against the folate receptor of tumor cells, KB cells were seeded onto cover glass in 6-well plates and cultured overnight in a 5% CO_2_ incubator (37°C) with RPMI 1640 media (supplemented with 10% FBS and 1% antibiotics). Cells were washed with PBS (pH 7.4, 0.1 M) and treated with 5 μg/ml of DOX or nanoparticles for 1 h. DOX was dissolved in DMSO and then diluted at least 100 times with folic acid free-RPMI1640 medium. Lyophilized nanoparticles were carefully reconstituted into folic acid free-RPMI1640 medium. To prove targetability of nanoparticles, KB cells were pre-treated with folic acid (5 mM) 1 h before addition of nanoparticles. After 1 h, cells were washed with PBS and treated with 4% paraformaldehyde. Cells were washed again with PBS and fixed by immobilization solution (ImmuMount, Thermo Electron Corporation, Pittsburgh, PA). These cells were observed by confocal laser scanning microscopy (CLSM) using a TCS-SP2 microscope (Leica, Wetzlar, Germany). Flow cytometry analysis of cells was also performed to assess targetability of nanoparticles. KB cells were seeded at a density of 1 × 10^6^ cells in 6-well plates and incubated overnight. Cells were treated with DOX or nanoparticles as described above. After 1 h, cells were harvested and analyzed with a flow cytometer. An excitation wavelength at 488 nm and emission wavelength at 522 nm were used to observe the fluorescence intensity of DOX.

### *In vitro* antitumor activity

Nanoparticle antitumor activity against KB cells were assessed by an established viability assay based on 3-(4,5-dimethylthiazol-2-yl)-2,5-diphenyltetrazolium bromide (MTT) *in vitro*. KB cells (3 × 10^4^) were seeded in 96-well plates and incubated overnight in a 5% CO2 incubator at 37°C. DOX or nanoparticles (DOX concentration, 50 μg/ml) were added to wells in the absence or presence of folic acid and then incubated for 4 h. The medium was replaced with a serum-free medium in the absence of folic acid. After 1 day, 25 μl of MTT (2 mg/ml) was added to the 96-well plates and incubated for 4 h. The formazan crystals formed in living cells were solubilized with a sodium dodecyl sulfate (SDS)/HCl solution (SDS, 10% *w*/*v*; HCl 0.1 N), and the absorbance was determined at 570 nm using an automated computer-linked microplate reader (Molecular Devices, Sunnyvale, CA, USA).

### *In vivo* fluorescence imaging of NIH3T3 and KB-tumor-bearing mouse

1 × 10^6^ KB cells and 1 × 10^6^ NIH3T3 cells were implanted onto the right side and left side of the back of nude BALb/C mice (5 weeks, 20 g), respectively. Three weeks later, NIR-dye-conjugated nanoparticles (dose: 20 mg/kg, injection volume: 100 μl) were injected intravenously (i.v.) into the tail vein of the mouse. At specific time intervals, the trace of nanoparticles in the mouse was observed with Maestro™ 2 small animal imaging instrument (Cambridge Research and Instruments, Inc., Woburn, MA, USA). After nanoparticle administration (48 h), the mice were sacrificed to observe organ distribution of nanoparticles, and each organ of the mice was also observed with Maestro™ 2 small animal imaging instrument. All animal studies were performed under guidelines of the Committee of Care and Use of Laboratory Animals of Chonnam National University.

### Statistical data analysis

The results are expressed as the means of three parallel experiments ± standard deviation. Statistical analysis of the results was performed using the t-test with *p* < 0.05 as the minimal level of significance.

## Results

### Characterization of FAPuLG copolymer

**Figure 1:**
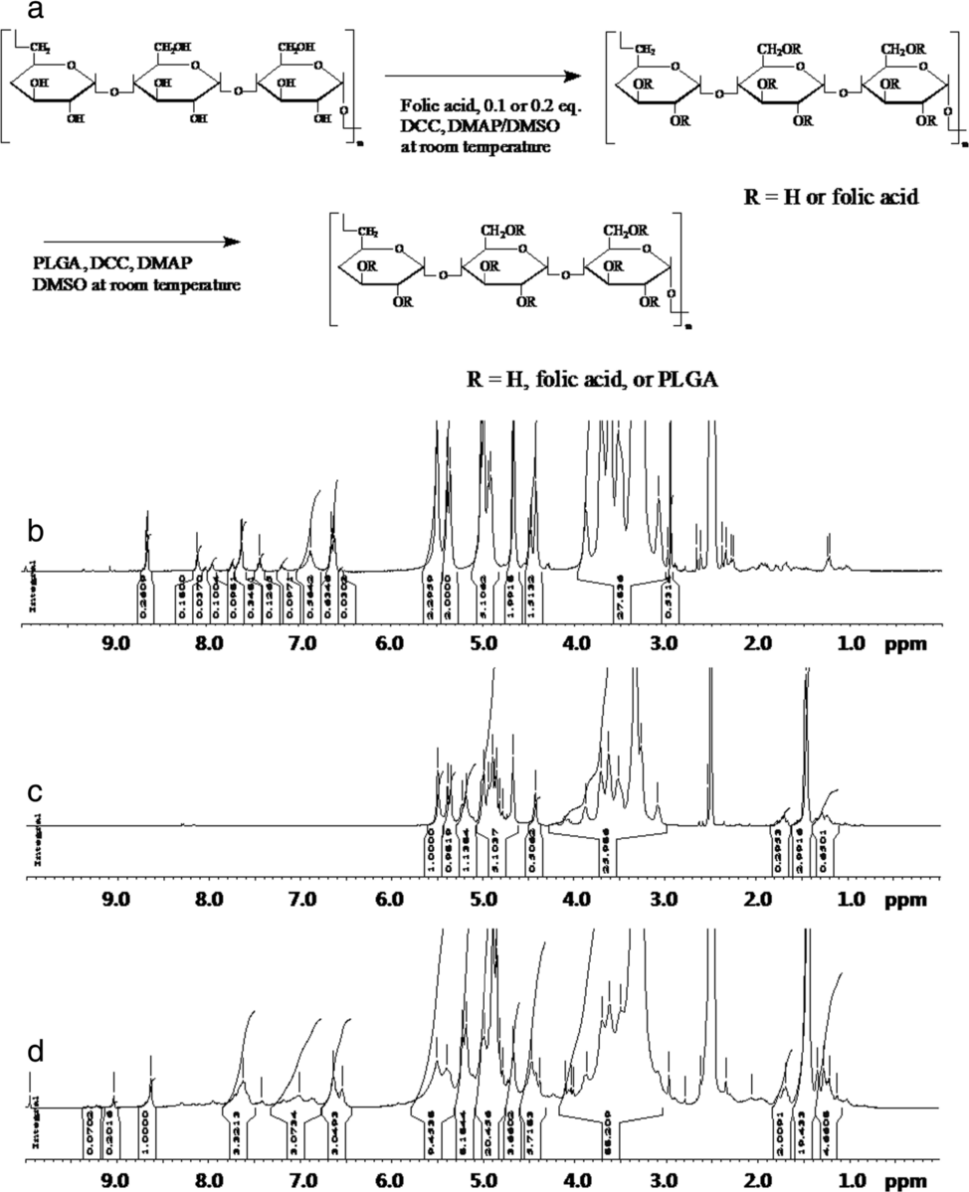
**Synthesis scheme of FAPuLG copolymer (a)** ^**1**^ **H NMR spectra of FA-PU (b), PuLG (c), and FAPuLG (d) conjugates.**

Figure 1a depicts the synthesis scheme of the FAPuLG copolymer. Folic acid was conjugated to hydroxyl groups of pullulan using carbodiimide chemistry. Specific peaks of pullulan appeared between 3.0 ~ 5.8 ppm, and specific peaks of folic acid appeared at 6.5 ~ 9.0 ppm (Figure 1b). The substitution degree of folic acid against 100 glucose units of pullulan backbone was 9.88 and 35.8, respectively (Table 1). PLGA was also conjugated to FA-PU conjugates (Figure 1). The methylene proton of PLGA was appeared at 1.5 ppm (Figure 1d). These results indicated that FAPuLG copolymers were successively synthesized. Furthermore, PuLG copolymer without folic acid was synthesized for comparison and its characteristic peaks of pullulan and PLGA (Figure 1c). The substitution degree of folic acid and PLGA was calculated by ^1^H NMR spectra from known M.W.s of pullulan and PLGA [17]. Table 1 shows the characteristics of FAPuLG copolymer. The substitution degree of PLGA for PuLG, FAPuLG-1, and FAPuLG-2 was 1.27, 1.46, and 1.78, respectively.

**Table 1:** **Characterization of FAPuLG copolymer**

**Polymer**	**Theoretical ratio** ^**a**^					**Experimental ratio** ^**c**^		
	**FA**	**PU** ^**a**^	**PLGA**	**M.W.**		**FA**	**PU**	**PLGA**
Pullulan	-	100	-	67,500*			100	
FAPu 1	10	100	-	86,244^b^	570 mg	9.88	100	
FAPu 2	20	100	-	105,411^b^	630 mg	35.8	100	
PLGA	-	-	100	8,200*				
PuLG	-	100	1.2	108,684^b^	250 mg	0	100	1.27
				106,696^c^				
FAPuLG 1	10	100	1.2	146,267^b^	260 mg	6.00	100	1.46
				151,453^c^				
FAPuLG 2	20	100	1.2	127,244^b^	210 mg	21.5	100	1.78
				139,918^c^				

*M.W. was evaluated from M.W. of pullulan and PLGA as reported previously [17]. ^a^The feeding molar ratio of FA and PLGA versus 100 glucose unit of PU; ^b^Mn - theoretical value; ^c^Mn was analyzed with NMR.

### Characterization of nanoparticles

**Figure 2:**
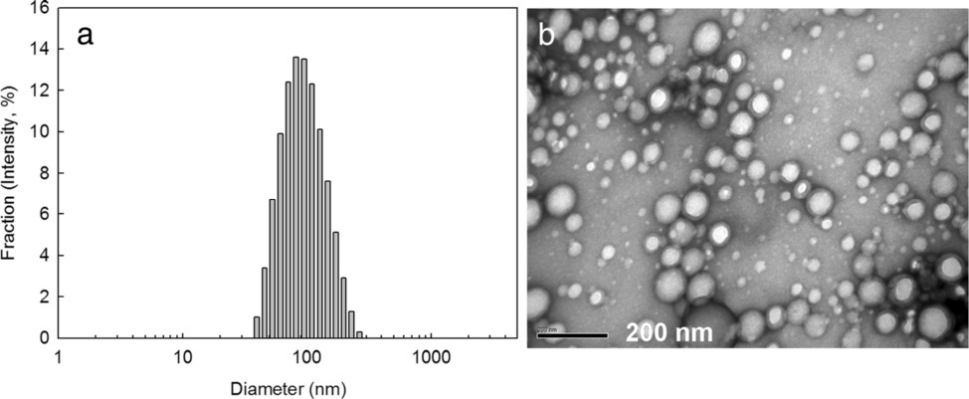
**Typical particle size (a) and TEM image (b) of FAPuLG-1 nanoparticles.**

**Figure 3:**
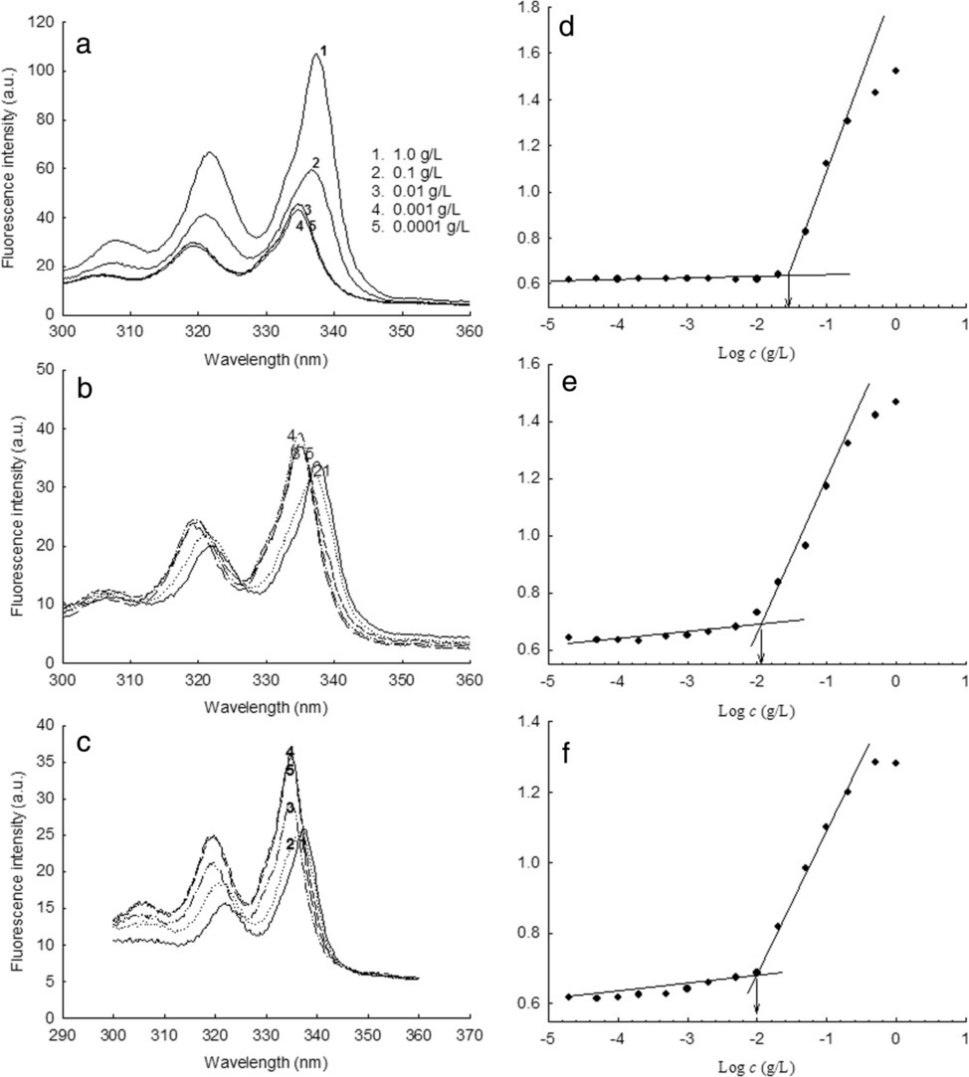
**Fluorence excitation and plots of intensity ratios.** Fluorescence excitation of pyrene (6.0 × 10 − 7 M) vs. the concentration of PuLG **(a)**, FAPuLG-1 **(b)**, and FAPuLG-2 **(c)** nanoparticles in distilled water (λem = 390 nm). Plots of the intensity ratios, I338/I335, from the pyrene excitation spectra vs. log c of the PuLG **(d)**, FAPuLG-1 **(e)**, and FAPuLG-2 **(f)** nanoparticles in distilled water. Each point was derived from the fluorescence spectra curve. The arrows indicate the signal changes in the crossover region.

**Figure 4:**
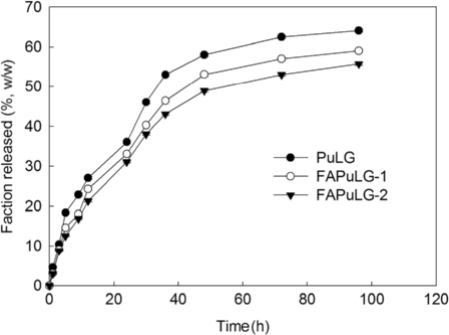
**DOX release from PuLG and FA-PuLG nanoparticles.**

To make nanoparticles, FAPuLG was dissolved in DMSO and then added dropwise to deionized water. Organic solvent was removed by dialysis method. The average diameter of the formed nanoparticles was consistently <200 nm (Figure 2a, Table 2). Particle sizes of FAPuLG were slightly larger than PuLG, suggesting that the folic acid moiety may increase their average diameter. Furthermore, PuLG and FAPuLG nanoparticles were spherical (Figure 2b). Their sizes were not significantly different from the results of particle size measurement. Since PuLG and FAPuLG are amphiphilic polymers composed of the hydrophilic domain of pullulan and hydrophobic domain of PLGA, formation of nanoparticles occurred by self-aggregation process in the aqueous environment. To study the self-assembly of nanoparticles, fluorescence spectroscopy measured CAC using pyrene as a fluorescence probe. The fluorescence spectra showed a red shift according to the increase of copolymer concentration, indicating that the self-assembly of the copolymer was formed in water, and then, pyrene was preferentially partitioned into the core of the nanoparticles (Figure 3a,c). A flat region and a sigmoid change in the crossover region were observed at low concentrations of copolymer (Figure 3d,f). These crossover regions were known to be the CAC of the copolymer. The CAC of PuLG, FAPuLG-1, and FAPuLG-2 was 0.026 g/l, 0.011 g/l, and 0.01 g/l, respectively. DOX was used as a model anticancer drug. DOX was incorporated into the PuLG and FAPuLG nanoparticles. The results are summarized in Table 2. When DOX was incorporated into the nanoparticles, sizes were increased significantly compared to empty nanoparticles. The drug release kinetics was shown in Figure 4. The drug release rate was relatively fast until 36 h, after which release was continuous. The nanoparticles having larger diameter and higher drug contents induced a slower release rate of drugs.

**Table 2:** **Characterization of FAPuLG nanoparticles**

	**CAC (g/l)**	**Average diameter (intensity, nm)**	**Drug contents (%,** ***w*** **/** ***w*** **)**	
			**Theoretical**	**Experimental**
Empty nanoparticles				
PuLG	0.026	87		
FAPuLG-1	0.011	94.4		
FAPuLG-2	0.01	116.8		
DOX-loaded nanoparticles				
PuLG		123.1	20	6.8
FAPuLG-1		168.1	20	7.2
FAPuLG-2		173.1	20	7.4

### Folate-receptor-mediated endocytosis and targeting of FAPuLG nanoparticles

**Figure 5:**
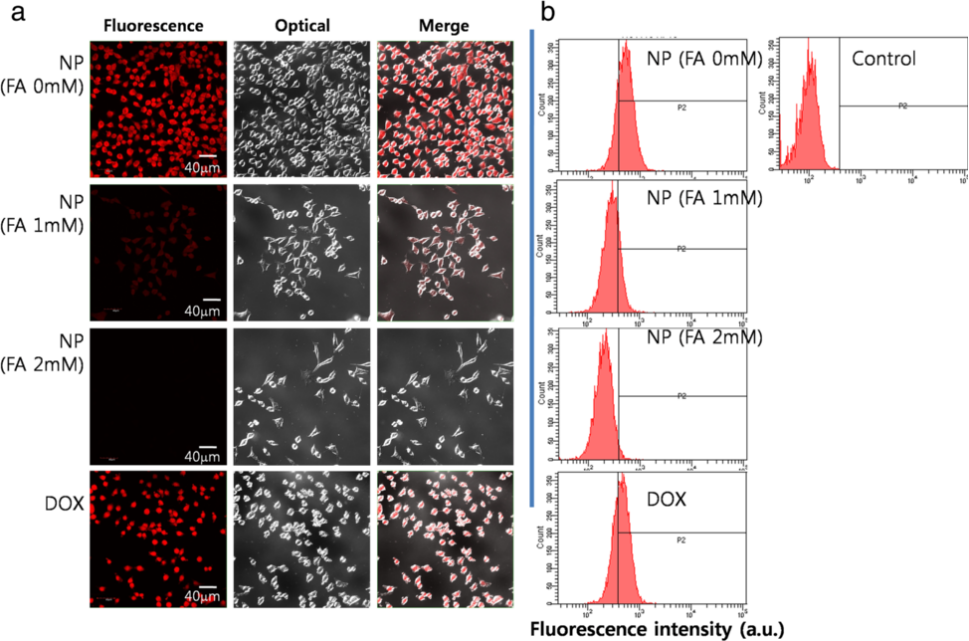
**Confocal laser scanning microscope images (a) and flow cytometric analysis (b).** KB cells were treated with DOX or FAPuLG-2 nanoparticles. For blocking of the folate receptor of tumor cells, folic acid was pre-treated 1 h before drug or nanoparticle treatment.

**Figure 6:**
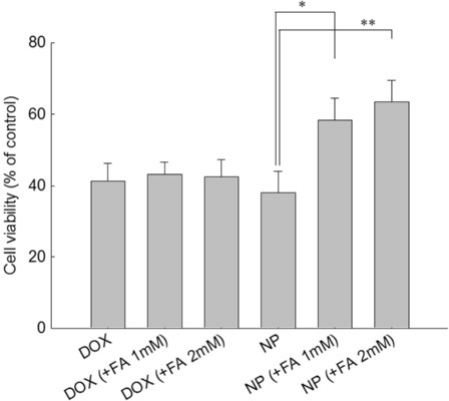
Cytotoxicity of DOX-incorporated FAPuLG nanoparticles against KB cells (*, ** = ***p*** **< 0.05).** KB cells were pre-treated with folic acid 1 h before drug treatment, and then, cells were exposed to 50 μg/ml of DOX or nanoparticles for 4 h. Twenty-four hours later, viable cells were determined using the MTT assay.

To test receptor-mediated delivery of drugs, DOX was used as a model drug because it has strong red fluorescence and can be used instead of a fluorescence marker. DOX-incorporated FAPuLG nanoparticles were studied using KB cells. Representative fluorescence images and flow cytometry results are depicted in Figure 5a,b, respectively. KB cells revealed strong red color when DOX or DOX-incorporated FAPuLG nanoparticles were treated, indicating that DOX or DOX-incorporated FAPuLG nanoparticles were entered into the tumor cells (Figure 5a). However, red fluorescence was decreased significantly when the folate receptor was blocked by pre-treatment with folic acid. These results indicated that nanoparticles of FAPuLG nanoparticles entered into the cells through folate-receptor-mediated endocytosis. Flow cytometry analysis also supported these results (Figure 5b), indicating that fluorescence intensity of KB cells was increased when DOX or DOX-incorporated nanoparticles were treated. Fluorescence intensity was decreased by the blocking of the folate receptor. Cell viability was checked to test receptor-mediated delivery of drug, and then, nanoparticles can selectively kill the tumor cells (Figure 6). Cell viability of FAPuLG nanoparticle treatment did not differ with the DOX values. However, viability of cell populations treated with FAPuLG nanoparticles increased when the folate receptor was blocked with folic acid. However, cell viability with DOX treatment was not significantly changed by blocking of folate receptor. These results indicated that FAPuLG nanoparticles can target the folate receptor of KB cells and selectively kill tumor cells.

**Figure 7:**
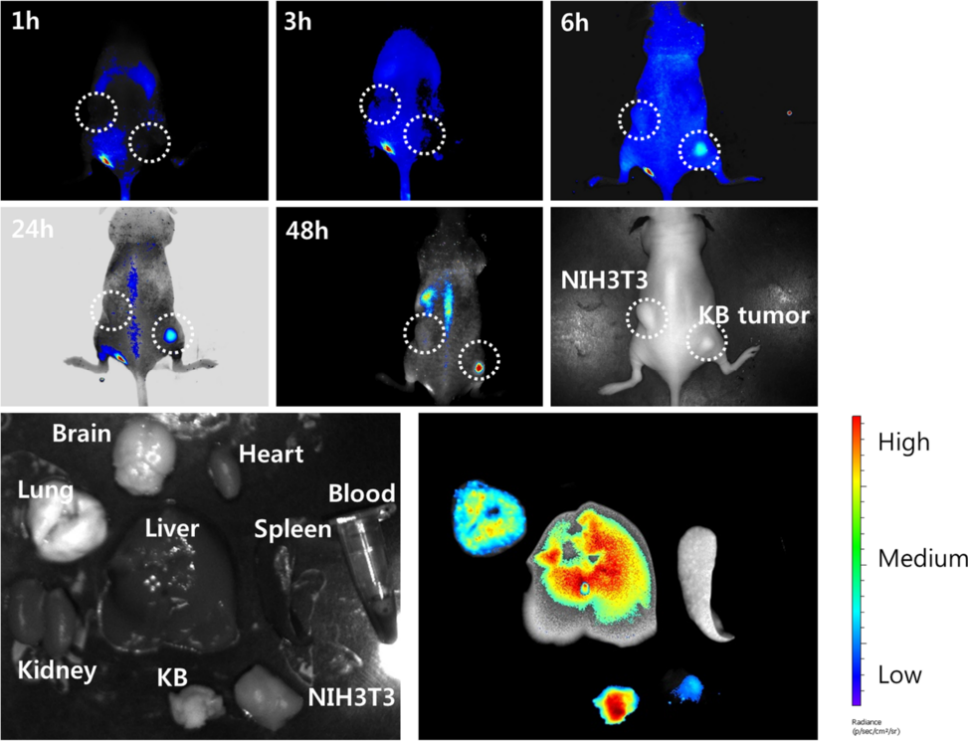
**The NIR fluorescence imaging study.** BALb/C nude mouse bearing an NIH3T3 (left side) and KB tumor (right side) was used. NIR-dye-conjugated nanoparticles (20 mg/kg) were intravenously injected via the tail vein of the mouse. The major organs of the mice were taken 48 h after administration. The data shown as the mean ± SD (*n* = 4).

For *in vivo* biodistribution imaging of FAPuLG nanoparticles, FAPuLG was conjugated with water-soluble NIR dye. To investigate KB tumor cell specificity of NIR-dye-conjugated FAPuLG, KB cells (folate-receptor-positive tumor cell) and NIH3T3 cells (folate-receptor-negative mouse fibroblast cells) were simultaneously implanted to the right side and left side of the nude mice as shown in Figure 7. Fluorescence intensity was continuously strong in the right side of the mice until 48 h. Even though FAPuLG nanoparticles were also considerably accumulated in the liver at organ distribution, FAPuLG nanoparticles in the solid mass of KB tumor were significantly higher than the solid mass of NIH3T3. These results indicated that FAPuLG nanoparticles have specificity for the KB cells and targetability against folate receptor of the tumor cells.

## Discussion

Targeted drug delivery using nanoparticles is a promising strategy for treatment of tumor cells [1-13]. Since tumor cells generally over-express various kinds of receptors on the cell membrane, receptor-mediated delivery of bioactive agents is an ideal way to maximize antitumor activity and minimize the unwanted side effect of anticancer drugs. Nanoparticles are an ideal candidate for these purposes because the target ligand can easily be decorated on the large surface area of nanoparticles. Nanoparticles have the unique feature of a small size (around 100-nm diameter), which enables nanoparticle virus-like behavior and passive targeting in the human body [18]. Various target ligands have been developed to decorate the surfaces of colloidal carriers and nanoparticles [5-10]. Among them, folic acid is often selected for tumor targeting of nanoparticulate anticancer drugs because most of tumor cells have numerous folate receptors on the cell surface [8]. Then, targeting to the folate receptor of tumor cells enables nanoparticles to concentrate anticancer drugs in tumor cells and to avoid healthy cells. Zwicke et al. introduced the folate receptor for active targeting of cancer cells using various kinds of nanomaterials [8]. Folic-acid-decorated nanomaterials can be used to various kinds of cancer therapies, such as cancer chemotherapy, cancer imaging, and/or imaging of circulating cancer cells in the human body [8,18,19].

In this study, we used pullulan for conjugation of folic acid and fabrication of nanocarriers of the anticancer drug, DOX. Pullulan, which is a water-soluble, neutral, linear polysaccharide, has properties of biocompatibility and biodegradability. These attributes have been exploited in the extensive biomedical use of pullulan as a nanomaterial [17,20-22]. Since pullulan is highly water-soluble, copolymerization with PLGA endowed amphiphilic properties to copolymers, i.e., pullulan backbone acts as a hydrophilic domain and PLGA acts as a hydrophobic domain [17]. PLGA can form a hydrophobic drug container while pullulan is normally exposed on the surface of nanoparticles. Furthermore, folic acid conjugated with a pullulan backbone also can be exposed on the surface of nanoparticles due to amphiphilicity of FAPuLG copolymer and then used as a targeting ligand for cancer targeting. Figure 4 depicts the self-assembly property of the FAPuLG copolymer in an aqueous environment and the resulting very low CAC values. PuLG and FAPuLG nanoparticles have small particle sizes <200 nm, which allow drug targeting to specific organs and tissues, avoidance of the reticuloendothelial system, prolonged blood circulation, and virus-like behavior [23]. FAPuLG nanoparticles showed targetability against the folate receptor of KB cells (Figure 5), and antitumor activity of DOX was controlled by folate-receptor-mediated drug delivery, indicating that nanoparticles of FAPuLG can be used to deliver an anticancer agent specifically to the folate receptor of tumor cells. Watanabe et al. also reported that folate-polymer conjugate can target the folate receptor of KB cells, with the IC_50_ of folate-polymer conjugate-coated liposomes being twofold lower than polymer liposomes [24]. We also obtained positive results from the anticancer activity of FAPuLG nanoparticles (Figure 6). DOX-incorporated nanoparticles retained still cytotoxicity against tumor cells even though the folate receptor was blocked. These results might be due to the passive diffusion of drug or nanoparticles into the cells. However, cell viability with treatment of FAPuLG nanoparticles in the presence of folic acid was increased 20% higher than that in the absence of folic acid, indicating that FAPuLG is a promising candidate for active targeting of the folate receptor of tumor cells. Furthermore, we demonstrated that FAPuLG nanoparticles have targetability against KB tumor as shown in Figure 7. Fluorescence imaging study of animals using NIR-dye-conjugated nanoparticles showed that fluorescence intensity was significantly higher in the solid mass of KB tumor cells than in the solid mass of NIH3T3. These results indicated that FAPuLG nanoparticles can specifically target the folic acid receptor of tumor cells. Shen et al. also reported that folic-acid-decorated nanophotosensitizer was highly sensitive for targeting of the folate receptor of KB cells but not for NIH3T3 normal cells [25]. Furthermore, they also described that the viability of NIH3T3 normal cells was not affected by the treatment of nanoparticles regardless of folic acid decoration while viability of KB cancer cells was sensitively decreased according to the presence of folic acid on the nanoparticle surface. Ultimately, folic acid decoration onto the nanoparticles efficiently enhances drug targeting against cancer cell and tumor tissue *in vitro*/*in vivo*. We suggest FAPuLG nanoparticles as an ideal candidate for tumor targeting.

## Conclusions

In conclusion, we synthesized FAPuLG copolymer and folic-acid-decorated nanoparticles for active tumor targeting. Nanoparticles of FAPuLG copolymer have a small diameter (<200 nm) and targetability to the folate receptor of KB cells. We suggest that FAPuLG copolymer nanoparticles are promising vehicles for drug targeting.
